# Progressive Scoliosis in a Child with Cystic Fibrosis

**DOI:** 10.1155/2019/1471879

**Published:** 2019-04-10

**Authors:** Lauren M. Castner, Samya Z. Nasr, Manuel Arteta

**Affiliations:** Division of Pediatric Pulmonology, C.S. Mott Children's Hospital, University of Michigan, Ann Arbor, MI, USA

## Abstract

We discuss an adolescent female with cystic fibrosis, asthma, and scoliosis who had a rapid decline in her pulmonary function despite typical treatment for a cystic fibrosis exacerbation. Ultimately, she had a fixed airway obstruction likely due to her progressive scoliosis, which improved following surgical intervention.

## 1. Introduction

Cystic fibrosis (CF) is the most common autosomal recessive genetic disorder in Caucasians, and it affects multiple body systems [1]. It is associated with mucus retention and plugging, chronic infection, and airway inflammation that can lead to bronchiectasis, small airways obstruction, and progressive respiratory impairment [1].

Scoliosis is estimated to be present in 2-3% of children [2]. Adolescent scoliosis is further defined as scoliosis that begins after the age of ten years and does not have an associated underlying disease [3]. There can be complications of back pain, poor body image, and impaired pulmonary function in those with idiopathic scoliosis [2]. There are complex three-dimensional changes in the flexion and rotation of the thoracic vertebrae [4]. These changes alter the shape of the chest which may decrease the range of motion of the thoracic cage and spine during breathing, decrease the compliance of the chest wall, and place the respiratory muscles at a biomechanical disadvantage [4], which can all lead to pulmonary restriction. Restrictive lung disease (RLD) is common for those with progressive scoliosis and is associated with a decrease in lung capacity as the spinal curvature and Cobb angle increases [2] due to the changes in thoracic cage, chest wall, and respiratory muscles.

There is growing evidence that scoliosis is also associated with obstructive lung disease. Preoperative pulmonary function testing (PFT) with spirometry and lung volumes on patients with scoliosis has revealed restrictive, obstructive, and mixed ventilatory defects [2]. Kumar et al. have also performed a retrospective study on patients with CF and found that scoliosis has a prevalence of 15.6% in school age children, which is 20 times higher than children with similar demographics. The curves themselves were single-thoracic and short which were considered benign [5].

The following case presents an adolescent patient with CF who had progressive worsening of pulmonary function in the setting of scoliosis in a short period of time.

## 2. Case History and Examination

A 13-year-old female with a history of pancreatic insufficient CF and asthma developed 2-3 weeks of progressively increasing yellow, nasal drainage, sore throat, congestion, productive cough, fatigue, and decreased appetite. In addition to increasing her airway clearance regimen from twice daily to four times daily, she was started on Bactrim DS, 1 tablet twice daily for a possible MSSA sinusitis and CF pulmonary exacerbation. Because of persistent symptoms, spirometry was performed for further evaluation.

Spirometry demonstrated steady decline in pulmonary function over the last 6 months that was concerning for a more indolent pulmonary process. The patient was admitted for IV antibiotics and bronchoscopy. On physical examination, her initial vital signs revealed blood pressure 95/50 mmHg, heart rate 112 bpm, temperature 36.4°C, respirations 20 breaths per minutes, and SpO_2_ 97% on room air. She had a BMI at the 79^th^ percentile based on the CDC 2–20 year-old female BMI-for-age data. She was a well appearing, well nourished young woman who had normal work of breathing but faint crackles at the bases and the right lung apex. Her heart had a regular rate and rhythm, no murmurs, rubs, or gallops. Her abdomen was soft, nontender, nondistended without hepatosplenomegaly. Cranial nerves II-XII were grossly intact with 5/5 strength for hip flexion, ankle plantar/dorsiflexion, hand grip, and biceps extension. When a thorough spinal examination was performed, she had right thoracic prominence on an Adam's bend test with elevation of the right shoulder and slight asymmetry of her waist line.

## 3. Differential Diagnosis

In a patient with cystic fibrosis complaining of progressively worsening nasal drainage, cough, and fatigue, the differential includes a cystic fibrosis pulmonary exacerbation [6], which could be bacterial or viral in nature [7]. She had started treatment with an increased airway clearance regimen in order to reduce mucus plugging and was treated with oral antibiotics that were targeting her usual pulmonary microbiota. Due to her continued symptoms on outpatient therapy, her pulmonary exacerbation could have been more significant and she failed outpatient therapy [7], or another process could be occurring.

Her symptoms also are consistent with sinusitis. Nearly all patients with cystic fibrosis develop chronic sinus symptoms during their disease [8]. Again, thick mucus, impaired mucociliary clearance, and chronic infection and inflammation are predisposing factors to developing chronic sinus disease [8]. She had been started on culture-directed therapy with oral antibiotics, but remained symptomatic which again would be consistent with a significant infection or another process.

A pulmonary exacerbation with a previously unidentified organism or atypical organism could be the next differential for a patient with cystic fibrosis who is not improving with antibiotic therapy [7]. Patients with cystic fibrosis have an increased risk of treatment failure when they are chronically infected with *P. aeruginosa*, *B. cepacia* complex, nontuberculous mycobacteria, or MRSA or have allergic bronchopulmonary aspergillosis [7]. Children, especially young children, are unable to expectorate sputum for culturing, and lower respiratory tract microbiology is therefore assessed with oropharyngeal swabs. These swabs are generally inadequate in the detection and isolation of fungi, nontuberculous mycobacteria, and *Burkholderia cepacia* complex [9]. They are also not as sensitive for *S. aureus* and *P. aeruginosa* and are likely to contain oral flora [10]. Sputum samples may also not be as accurate as bronchoalveolar lavage fluid since it is the deepest sample [10].

Finally, it was felt that an airway abnormality could be contributing to her symptoms. The patient had never had a bronchoscopy; therefore, it was unknown if there were any airway abnormalities such as bronchomalacia. It was felt to be of lower likelihood due to the acute illness and progressive nature declining pulmonary function tests since bronchomalacia is a fixed obstruction. Mucus plugging would be more likely to be present due to her underlying disease.

## 4. Investigation

**Figure 1:**
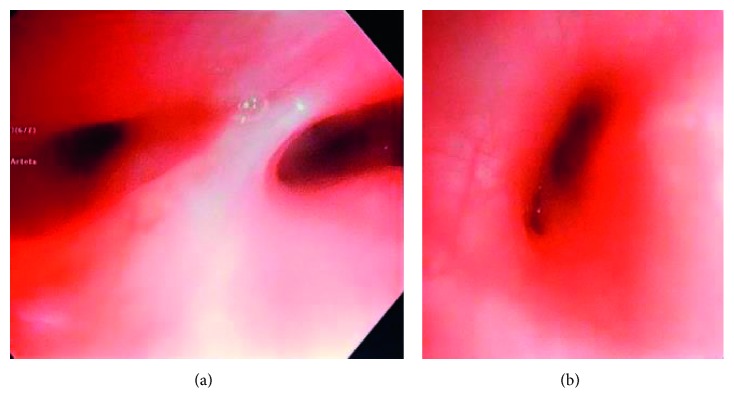
Bronchoscopic images of the narrowing of the subsegments of the right middle lobe (a) and right anterobasal segment (b).

The patient underwent a flexible bronchoscopy at the time of her admission. It showed an elongated narrowing of the bronchus intermedius extending into the right middle lobe take off and severe collapse of the right anterior basal segment bronchus preventing passage of the bronchoscope. There was no improvement when positive pressure ventilation was applied (Figure 1).

**Figure 2:**
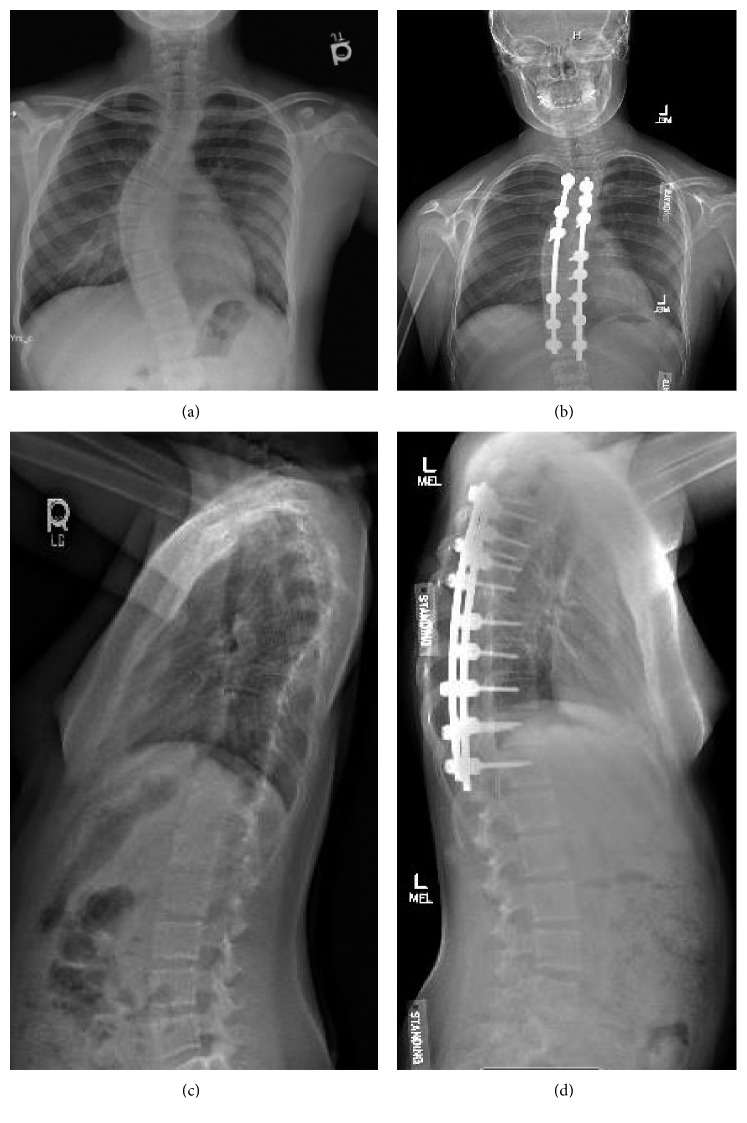
(a, c) Chest radiograph from admission for pulmonary exacerbation, October 4, 2016. (b, d) Spinal series six months after posterior spinal fusion, June 29, 2017.

Her initial chest X-ray revealed mild prominence of the central bronchial markings along with dextroconvex mid-thoracic curvatures, which had increased from previous images taken 2 months prior. A spinal series for scoliosis was performed and revealed a 35-degree levoconvex curve from T1 to T4 and a 60-degree dextroconvex curve from T5 to T11. There was no significant pelvic tilt or truncal shift, but there was some straightening of the spine in the lateral projection (Figure 2).

The bronchoalveolar fluid was positive for only oral flora and *Penicillium* species. A viral panel had also been performed which was positive for a human rhino/enterovirus infection. Otherwise, there were no other significant findings on cultures for acid-fast bacilli or the BAL pathology.

## 5. Treatment and Outcome

The patient was started on intravenous cefazolin to cover her previous MSSA cultures. She also started aggressive airway clearance with inhaled albuterol and chest physiotherapy four times daily. Orthopedics were contacted and recommended outpatient follow-up when she had recovered from her acute illness in order to perform a more thorough examination. After some initial improvement, the patient was discharged home after four days to complete a 21-day course of IV cefazolin and follow-up with orthopedics.

At her orthopedic follow-up 3 weeks following admission, it was recommended that she undergo posterior spinal fusion from T4–T12 since her spinal curve was greater than 50 degrees. She underwent posterior spinal fusion from T4–T12 with O-ring and her pulmonary function tests have made gradual improvements. Her FEV1 has increased from 64% predicted to 73% (Figure 1). Her spinal curvature measures have significantly improved as well: 30-degree levoconvex curvature from T1 to T5 and 28-degree dextroconvex curvature from T5 to L1. There was a modest change in her lateral alignment (Figure 2).

## 6. Discussion

Abnormal PFTs are common finding in adolescents with CF. Our patient had restricted lung disease with progressive worsening in the setting of scoliosis in a short period. While restrictive lung disease is a known complication of scoliosis, she had moderate to severe bronchial obstruction. This obstruction is probably due to direct external compression from the deviated thoracic spine, or rotation and distortion of the airway due to the altered thoracic anatomy [4].

Our patient did not recognize the symptoms arising from the deterioration in pulmonary function, likely due to the underlying CF symptoms. The trend of her pulmonary function showed that her scoliosis was leading to airway obstruction with improvement after spine surgery. It is known that there can be an immediate decline in PFTs immediately after surgery for scoliosis [11] and that it can take nearly 1-2 months before pulmonary function returns to baseline [11]. Our patient's pulmonary function followed this trend and then continued to improve.

**Figure 3:**
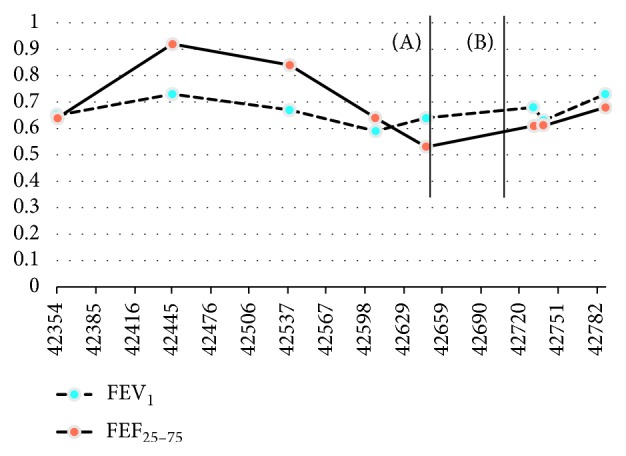
Trends in FEV_1_ and FEF_25–75_ from February 15, 2015, to February 22, 2017. Line A: time of admission and bronchoscopy; Line B: time of posterior spinal fusion. FEV_1_: forced expiratory volume in first second; FEF_25–75_: forced expiratory flow.

This case demonstrates the importance of further investigation when a patient has persistent and rapidly progressive symptoms. Our patient had a 31% decline in her FEF_25–75_ within three months' time and 9% decrease in FEV_1_ from her best test 6 months prior, along with rapid onset of symptoms not responsive to airway clearance and antibiotics. Flexible bronchoscopy is a valuable tool when a patient with CF has persistent symptoms or worsening pulmonary function despite appropriate therapy (Figure 3). In our patient's case, bronchoscopy helped demonstrate the localized nature of bronchial obstruction. A limitation to this case is the absence of a preoperative and postoperative CT scan of the chest and airways. This allows one to view the airways in relationship to the spine and determine where the obstruction is localized. This can be confirmed and evaluated with PFTs and bronchoscopy. Our patient only had a preoperative bronchoscopy and her PFTs were followed which prevents us from best understanding the relationship between her scoliosis and the airflow obstruction. Patients with cystic fibrosis undergo routine screening with pulmonary function and chest imaging to assist in the management of their pulmonary disease, yet it is essential to also continue evaluating for other disease processes especially in the pediatric population who is undergoing significant growth and developmental changes and may be at risk for other disease sequelae.

## Abbreviations

CF: Cystic fibrosis
RLD: Restrictive lung disease
PFT: Pulmonary function testing
FEV_1_: Forced expiratory volume in first second
FEF_25–75_: Forced expiratory flow.
